# Multiple synaptic and membrane sites of anesthetic action in the CA1 region of rat hippocampal slices

**DOI:** 10.1186/1471-2202-5-52

**Published:** 2004-12-03

**Authors:** Sky Pittson, Allison M Himmel, M Bruce MacIver

**Affiliations:** 1 Department of Anesthesia, Stanford University School of Medicine, SUMC S288, Stanford, CA 94305-5117; 2 Medical Student, University of California, San Francisco, CA 94143

## Abstract

**Background:**

Anesthesia is produced by a depression of central nervous system function, however, the sites and mechanisms of action underlying this depression remain poorly defined. The present study compared and contrasted effects produced by five general anesthetics on synaptic circuitry in the CA1 region of hippocampal slices.

**Results:**

At clinically relevant and equi-effective concentrations, presynaptic and postsynaptic anesthetic actions were evident at glutamate-mediated excitatory synapses and at GABA-mediated inhibitory synapses. In addition, depressant effects on membrane excitability were observed for CA1 neuron discharge in response to direct current depolarization. Combined actions at several of these sites contributed to CA1 circuit depression, but the relative degree of effect at each site was different for each anesthetic studied. For example, most of propofol's depressant effect (> 70 %) was reversed with a GABA antagonist, but only a minor portion of isoflurane's depression was reversed (< 20 %). Differences were also apparent on glutamate synapses-pentobarbital depressed transmission by > 50 %, but thiopental by only < 25 %.

**Conclusions:**

These results, in as much as they may be relevant to anesthesia, indicate that general anesthetics act at several discrete sites, supporting a multi-site, agent specific theory for anesthetic actions. No single effect site (e.g. GABA synapses) or mechanism of action (e.g. depressed membrane excitability) could account for all of the effects produced for any anesthetic studied.

## Background

General anesthetics have been shown to depress neuronal responses in virtually all brain areas studied and this depression has been proposed to result from actions at GABA_A_-mediated inhibitory synapses and postsynaptic chloride channels [1-4], potassium channels [5-7], or calcium channels [8-11], and/or at glutamate-mediated excitatory synapses [12-17]. The last decade has seen a major shift in our understanding of general anesthetic mechanisms of action, away from a non-specific Unitary theory of action, towards a detailed view of anesthetic actions at membrane receptor and ion channel targets for these agents [18,19]. It is likely that several anesthetic actions occurring at independent sites contribute in additive ways to depress neuronal circuits in higher brain structures. Alternatively, anesthetic effects could result from actions at only a few sites and this should become evident by studying overall effects on the CA1 neural circuit and 'chasing down' the underlying actions. In the present study, the effects produced by five general anesthetics were studied at several possible sites of action within the well characterized Schaffer-collateral to CA1 neuron circuit using electrophysiological recordings from rat hippocampal slices. The CA1 circuit has previously been shown to be depressed by anesthetics from several chemical classes [20-26] at concentrations which alter hippocampal electrical activity in chronically instrumented rats during anesthesia [27-29]. The five agents chosen for this study are all clinically used anesthetics and provide a good representation from unique chemical classes: a halocarbon (halothane), halogenated ether (isoflurane), barbiturate (pentobarbital), sulfonated-barbiturate (thiopental), and a newer di-isopropylphenol compound, propofol.

## Results and discussion

### Anesthetics enhance GABA-mediated inhibition

**Figure 1:**
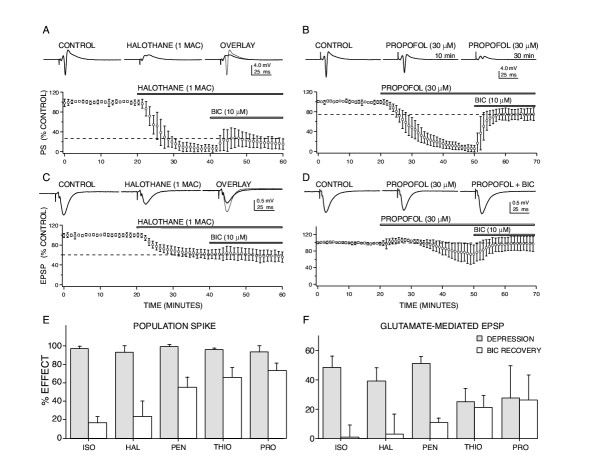
Anesthetic-induced depression of CA1 neuron responses involve actions at both glutamate and GABA-mediated synapses. (A) Halothane depressed population spike (PS) responses at clinically relevant concentrations (1 rat MAC = 1.2 vol % ~ 250 μM) and this depression was only partially reversed using a GABA_A _receptor antagonist, bicuculline (BIC). (B) Propofol (30 μM) produced a comparable degree of population spike depression compared to halothane, but this depression was substantially reversed with BIC, indicating that enhanced GABA-mediated inhibition contributes > 75 % of the depressant effect of propofol. The representative recordings shown are for propofol effects at 10 minutes following exposure to anesthetic (i.e. at 30 min on the x axis for the grouped data) and after 30 minutes of exposure, when a nearly complete block of the population spike was apparent. (C) Excitatory postsynaptic potentials (EPSP) were also depressed by halothane and this effect was not reversed by BIC, indicating a direct effect of the anesthetic on glutamate-mediated synapses. (D) Propofol-induced depression of glutamate-mediated EPSPs, in contrast, appeared to involve enhanced GABA-mediated inhibition, since this depression was completely reversed by BIC. The two anesthetics exhibited quite different sensitivities to reversal by BIC, indicating that actions at GABA synapses vary for these agents. For each graph, data were normalized and each point represents the mean ± SD for at least five measures from different slices made from separate animals. Horizontal bars indicate the time of exposure to each drug. Sample recordings from representative experiments are shown in the top traces. (E) BIC reversal of anesthetic-induced population spike depression was agent specific for equieffective levels of depression, and data are summarized for four anesthetics as bar graphs. Shaded bars represent the degree of depression produced by each anesthetic and open bars show the extent of reversal produced by BIC, error bars indicate SD for at least five measures from different slices. Volatile agents (isoflurane – ISO, 350 μM; halothane – HAL, 250 μM) were only weakly reversed by BIC, while intravenous agents (pentobarbital – PEN, 400 μM; thiopental – THIO, 80 μM; propofol-PRO, 30 μM) were more sensitive to the GABA receptor antagonist. (F) A similar profile of agent specific effects for BIC reversal was evident for glutamate-mediated EPSP responses, volatile agent effects were poorly reversed while intravenous agents appeared to be more sensitive to the GABA antagonist.

All five anesthetics depressed synaptically evoked discharge, measured as a block of population spike (PS) responses recorded from CA1 neurons (Fig. 1). The two volatile anesthetics, halothane and isoflurane, produced a nearly complete depression (to 3.3 ± 3.5 and 5.6 ± 7.1 % of control respectively) at clinically effective concentrations: halothane (1.0 rat MAC; 1.25 vol % ~ 250 μM) and isoflurane (1.0 rat MAC; 1.55 vol % ~ 350 μM; for Sprague-Daley rats [30]; Minimum Alveolar Concentration – the expired anesthetic gas concentration for a 50 % loss of a tail clamp response – motor reflex in rats). The three intravenous agents, pentobarbital (400 μM), thiopental (80 μM) and propofol (30 μM), also depressed PS responses to a comparable degree: 1.7 ± 3.1, 3.4 ± 2.8 and 6.2 ± 5.8 % of control responses, respectively (p < 0.001, n ≥ 5 slices from individual rats, for all five agents compared with pre-anesthetic control responses, using ANOVA-Tukey). All anesthetic effects were reversible on washout of the agent with drug free ACSF. It should be noted that the more lipophilic intravenous anesthetics produce lower effect site concentrations in these brain slices than the applied concentrations shown, especially for these short time periods of application, because it can take several hours for these agents to diffuse 200 to 300 μm into brain slices and achieve steady-state levels [31]. For example, an applied concentration of 30 μM propofol would be expected to produce only ~ 1.0 to 3.0 μM at a recording depth of 250 microns within 30 minutes [32]. The volatile anesthetics, in contrast, rapidly equilibrate throughout the brain slice due to their relatively high aqueous solubility.

These equi-effective applied concentrations for PS depression were used in subsequent experiments to determine whether this depression resulted from enhanced GABA_A_-mediated inhibition.

A GABA_A _receptor antagonist, bicuculline, was applied in the continued presence of each anesthetic to attempt to reverse the anesthetic-induced PS depression. Bicuculline (10 μM) reversed anesthetic-induced PS depression to varying degrees for each agent: isoflurane – 16.2 ± 7.4 %, halothane – 22.3 ± 18.4 %, pentobarbital – 56.2 ± 12.4 %, thiopental – 64.9 ± 12.9 % and propofol – 69.5 ± 14.3 %. Similar degrees of reversal were observed using the GABA-chloride channel blocker, picrotoxin (100 μM; a supra-maximal blocking concentration). A GABA_B _receptor antagonist, CGP 55845A (10 μM) did not reverse PS depression for any of the anesthetics studied (see Table 1). None of the anesthetics produced a significant depression of antidromically stimulated PS responses (± 5 % depression, p > 0.15) indicating that CA1 neuron axonal conduction was not appreciably altered. Thus, enhanced GABA_A_-mediated inhibition appeared to play a major role for the PS depression produced by propofol and thiopental (~ 75 %), less so for pentobarbital (~ 50 %), and contributed only partially to the depressant effects of the volatile anesthetics (< 25 %; Fig. 1E).

### Anesthetics depress glutamate-mediated excitatory synapses

To determine whether anesthetic-induced PS depression resulted from depressed glutamate-mediated excitatory synaptic inputs to CA1 neurons, field excitatory postsynaptic potentials (EPSPs) were recorded from dendritic regions in stratum radiatum. All five anesthetics depressed EPSP responses (e.g. Fig. 1C and 1D): isoflurane to 52.2 ± 7.6 (p < 0.001), halothane 61.3 ± 8.4 (p < 0.001), pentobarbital 54.5 ± 4.8 (p < 0.001), thiopental 75.5 ± 9.8 (p < 0.01) and propofol 72.7 ± 23.5 (p < 0.05) % of control responses. Bicuculline did not reverse volatile anesthetic-induced EPSP depression, but did partially reverse the effect for pentobarbital (11.4 ± 3.6 %) and completely reversed the EPSP depression produced by thiopental and propofol (Fig. 1F). Thus, depressed glutamate-mediated synaptic excitation appeared to play an important role for PS depression produced by isoflurane, halothane and pentobarbital. The thiopental and propofol-induced EPSP depression would also contribute to PS depression for these agents, but appeared to occur via enhanced GABA-mediated inhibition at a dendritic level, since this depression was reversed by bicuculline.

### Pre- and postsynaptic sites of action at GABA_A _synapses

**Figure 2:**
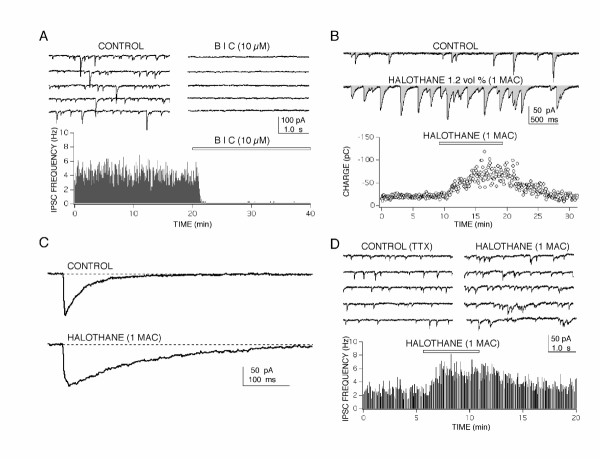
Whole cell patch clamp recordings of spontaneous GABA-mediated inhibitory postsynaptic currents (IPSC) in CA1 neurons revealed two sites of action for anesthetic-induced enhanced inhibition. (A) Spontaneous synaptic currents were blocked by a GABA_A_-receptor antagonist bicuculline (BIC), indicating that they were GABA_A_-mediated Cl^- ^currents. Traces on the top show 20 s of continuous recording from a CA1 neuron in control and in the presence of BIC. A rate meter graph (bottom) shows the relatively stable frequency of IPSCs during 20 min of control recording, followed by a rapid and complete block of responses produced by BIC (indicated by the bar). (B) Halothane produced a marked increase of the inhibitory charge transfer in CA1 neurons measured as the integral of current recordings (shaded area in top traces). A three fold increase of inhibitory charge (pico Colombs – pC) was reversibly produced by halothane and appeared to come about through both a prolongation of IPSC time course (C) and from an increased frequency of synaptic currents (D). Both effects persisted in the presence of 10 μM tetrodotoxin (TTX) and/or glutamate receptor antagonists indicating that action potential dependent activity and glutamate synapses were not required for anesthetic action. For the rate meter histograms in (A) and (D), each bin represents the number of events recorded in 4 s divided by 4 to give a frequency in Hz (events/second). For all IPSC recordings a CsCl based internal solution was used in the patch pipette.

Whole cell voltage clamp recordings from CA1 neurons were used to examine more closely anesthetic effects on membrane currents at GABA synapses. Spontaneous GABA-mediated inhibitory postsynaptic currents (IPSCs) were observed in all CA1 neurons studied (n = 15) and were completely blocked by bicuculline (10 μM; Fig. 2A). In the presence of glutamate receptor antagonists (CNQX 17.2 μM and APV 100 μM) the anesthetics produced agent-specific effects on holding currents needed to clamp neurons at the control resting membrane potentials (-60 to -70 mV). Propofol was most effective at increasing holding currents (376 ± 83 pA, n = 3), followed by thiopental (320 ± 72 pA, n = 4) and pentobarbital (127 ± 65 pA, n = 3). Halothane (n = 6) and isoflurane (n = 3) produced weaker and more variable responses (< 50 pA). The changes in holding currents produced by propofol and the barbiturates were reversed by bicuculline (10 μM) or picrotoxin (100 μM), indicating that they involved activation of GABA_A_-mediated chloride channels.

The most dramatic effect produced by all five anesthetics was observed on IPSCs (e.g. Fig. 2B). Membrane charge transfer, for example, was increased by 3 to 4 fold in the presence of halothane and came about by at least two separate mechanisms. The first mechanism was a prolongation of IPSC time course (Fig. 2C) resulting in nearly a 3 fold increase in charge transfer for each IPSC (284 ± 33 % of control, p < 0.005, n = 6). This result was in good agreement with previous findings showing that anesthetics prolong IPSCs by increasing the open time of GABA-gated channels in the postsynaptic membrane [33-35]. The second mechanism appeared to involve presynaptic sites, observed as an increase in frequency of IPSCs (143 ± 28 % of control, p < 0.005, n = 6 neurons from separate slices) and occurred with a small, but significant, depression in IPSC amplitudes (92 ± 6 % of control, p < 0.05, n = 6). The anesthetic-induced IPSC frequency increase was also observed in the presence of tetrodotoxin, used to block action potentials (n = 5 for halothane, n = 4 for propofol), indicating a direct action on GABA nerve terminals. This confirms earlier findings that anesthetics can increase IPSC frequency and the release of GABA from nerve terminals [36-39]. This presynaptic effect combines with postsynaptic prolongation of IPSCs to account for the marked increase in membrane charge transfer observed, and would contribute to the anesthetic-induced postsynaptic hyperpolarization of CA1 neurons previously reported [4,40-42]. All of the anesthetics studied increased inhibitory charge transfer and the degree of enhancement corresponded well with the ability of bicuculline to reverse the anesthetic-induced depression of population spike responses (Fig. 1 and Table 1). For halothane and isoflurane, this enhanced inhibitory charge transfer played a relatively minor role in population spike depression compared with their ability to depress glutamate-mediated excitatory inputs to the CA1 neurons.

### Anesthetics increase paired-pulse facilitation

**Figure 3:**
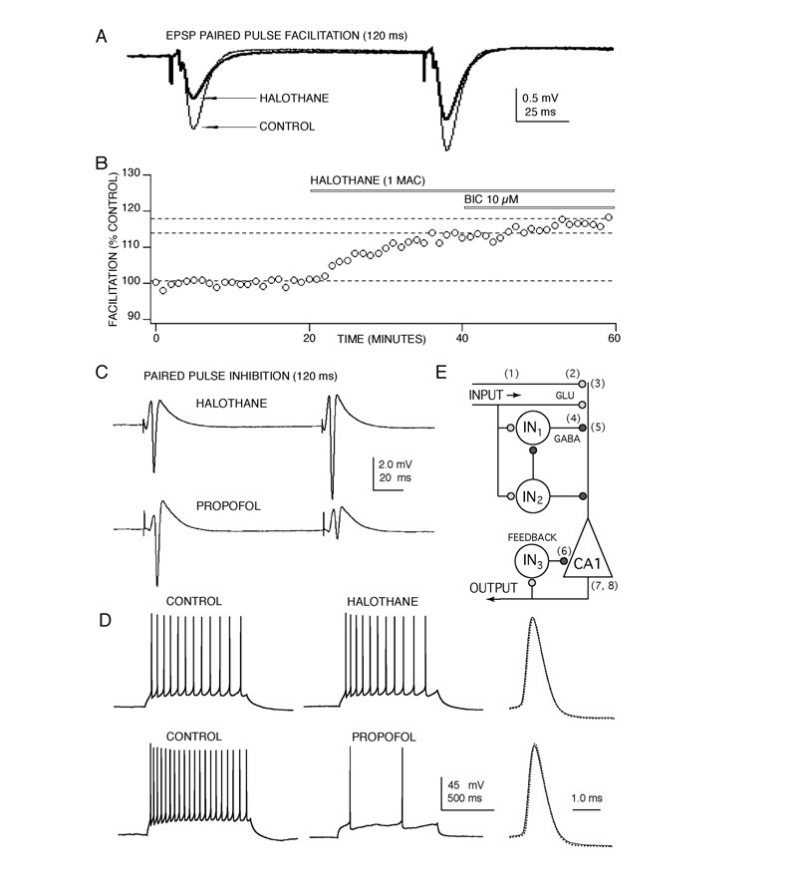
Anesthetics act at several sites to depress CA1 neuron synaptically evoked discharge. (A) Halothane appears to act presynaptically to depress glutamate release, evidenced by an increase in paired pulse facilitation concomitant with EPSP depression. A similar increase in facilitation was produced by isoflurane and pentobarbital, but not by thiopental or propofol. No change in EPSP rise or decay time was apparent in the presence any anesthetic. (B) The increased facilitation produced by halothane, isoflurane and pentobarbital was not reversed by bicuculline (BIC) indicating a depressant effect on glutamate nerve terminals – independent of anesthetic effects at GABA_A _receptors. (C) Differential GABA effects were also evident for paired pulse inhibition of population spike responses. Volatile agents like halothane produced little or no paired pulse inhibition at concentrations that produced a half maximal depression of first pulse responses. In contrast, propofol increased paired pulse inhibition and similar effects were observed with thiopental and pentobarbital. This increase in paired pulse inhibition was reversed by bicuculline indicating that these anesthetics enhanced recurrent GABA_A_-mediated inhibition. (D) The anesthetics also appeared to act directly on CA1 pyramidal neuron membrane excitability to slow action potential discharge activity, although the intravenous agents were much more effective compared to volatile anesthetics. None of the anesthetics produced an appreciable effect on individual action potential amplitude or time course (right: control – solid line; anesthetic – dotted, for halothane on top and propofol on bottom). (E) Anesthetics act at multiple sites to depress the CA1 neuron circuit. Sites of action are indicated on a diagram of CA1 circuitry showing input from Schaffer-collateral fibers, local inhibitory interneurons (IN) and a CA1 pyramidal neuron (triangle). Action potential propagation in Schaffer-collateral fibers (1) was depressed by ~ 15% by halothane and this contributes about 25 % to EPSP depression [60, 61]; see also [17]. This effect did not contribute to anesthetic-induced increases in facilitation, because no change in facilitation occurred when a comparable amount of action potential depression was produced by tetrodotoxin [61]. Further presynaptic depression at glutamate nerve terminals (2) was evident from the increased facilitation observed (Fig. 3A&3B) and there is also good evidence for postsynaptic depressant effects on both NMDA and AMPA receptors (3) [16, 19, 62, 63]. Anesthetics also act pre- and postsynaptically at GABA-mediated synapses (4, see Fig. 2) and can also increase tonic GABA-mediated inhibition by acting as GABA agonists in the absence of synaptically released GABA (5) [46-48]. Perisynaptic and extrasynaptic tonic GABA_A _receptors (7) also contribute to the postsynaptic depression produced by isoflurane [64] as well as thiopental and propofol [4, 65]. Enhanced recurrent inhibition (6) plays and important role for anesthetics *in vivo *[45] and strong effects were evident in the present study for propofol, thiopental and pentobarbital (Fig. 3C), similar to effects previously reported for halothane in hippocampal slices [26]. In addition, anesthetics also directly depress CA1 neuron excitability by blocking calcium channels and enhancing potassium currents contributing to hyperpolarization (8) and increased discharge thresholds [40, 41, 42, also Nishikawa, Beida & Maclver, unpublished]. These latter effects could influence CA1 neuron discharge activity for near threshold responses, but for the stronger stimuli used in the present study, effects on GABA and glutamate synapses and on postsynaptic receptors for these transmitters appear to contribute most (~ 80 %) to the depressant actions observed.

To determine whether presynaptic actions also contribute to anesthetic effects at glutamate synapses, paired pulse (120 ms) facilitation of Schaffer-collateral evoked EPSPs were studied. In the presence of either halothane or isoflurane no apparent change in EPSP rise time or decay kinetics were observed (Fig. 3A), contrasting with the marked prolongation of IPSC decay time produced by the anesthetics. Facilitation was increased to nearly 115 % of control and this effect was independent of GABA_A_-mediated actions, since they persisted in the presence of the antagonist – bicuculline (Fig. 3B). This increase in facilitation is consistent with a presynaptic depression of glutamate release from nerve terminals, perhaps via depressant actions on voltage activated calcium or sodium channels which couple axon spike depolarization to the release of transmitter [8,9,17,43,44].

### Anesthetics increase paired-pulse inhibition

Agent-specific effects were observed for paired pulse inhibitory responses (120 ms separation) recorded from CA1 neurons (Fig. 3C). Halothane and isoflurane produced no apparent change in paired pulse responses, both the first and second population spike following a pair of stimuli were depressed to a similar degree by these anesthetics. In contrast, propofol, thiopental and pentobarbital increased paired pulse inhibition, evident in a greater degree of depression for the second of a pair of responses. To quantify these increases in paired pulse inhibition, effects on second pulse responses were compared at concentrations that produced a half maximal depression of first spike responses. At a level of 50 % depression of first pulse responses, pentobarbital produced a 134 ± 8 % increase in second pulse inhibition, thiopental produced a 156 ± 15 % increase and propofol produced a 149 ± 13% increase (p < 0.001, n = 5 for each agent compared to first pulse responses). This effect is consistent with *in vivo *findings [45] and is thought to reflect a greater degree of GABA-mediated inhibition contributing to the second of a pair of stimuli, via recurrent (feedback) activation of inhibitory interneurons caused by the first pulse [26].

### Anesthetics depress CA1 neuron excitability

To determine whether the anesthetics could alter postsynaptic membrane excitability, effects on action potentials evoked by direct current injection into CA1 neurons were studied. Differences in effect were apparent across anesthetic agents – hardly any effect was evident for halothane and isoflurane, but the barbiturates and propofol produced a significant depression of action potential discharge (Fig. 3D). When measured as a reduction in the number of action potentials produced in response to a one second long depolarizing current step, halothane produced an 8.2 ± 2.2 % depression and isoflurane an 11.6 ± 6.1 % depression (p < 0.01 for both agents compared to control responses). Propofol was much more effective at depressing CA1 discharge, producing a 93.5 ± 6.1 % depression (p < 0.001). Thiopental produced a 90.3 ± 9.9 % depression and pentobarbital a 79.5 ± 7.4 % depression (p < 0.001 for both anesthetics compared with control). The anesthetic-induced depression of spike discharge activity was accompanied by decreases in membrane resistance and to a lesser extent by small changes in membrane resting potential. In spite of the marked depressant effects observed for the intravenous anesthetics on spike discharge, none of the anesthetics appeared to alter action potential amplitude, rise time or decay profiles (Fig. 3D), suggesting that the major depressant effect was accounted for by actions on spike threshold – not on the sodium currents which underlie action potentials *per se*.

## Conclusions

Two conclusions can be drawn from these results: 1) for a given anesthetic, like halothane, multiple sites of action contributed in an additive manner to produce an overall depression of transmission through the CA1 neuronal circuitry (Fig. 3E); 2) for each anesthetic the degree of effect was agent specific at some of these sites. Together the results support a Multisite Agent Specific (MAS) mechanism of action for general anesthetics. This represents a departure from traditional Unitary theories of action in several important respects. Unitary theories posit that all anesthetics act via a common molecular mechanism, such as to change the fluidity of nerve cell membranes, or to enhance a potassium current, or most recently to enhance GABA-mediated inhibition [2,3]. With the MAS theory, no common site of action is required (nor apparent) for anesthetics. This is consistent with observations at the molecular, [46-50] cellular [22,51] and behavioral levels [52-55].

Differing degrees of action (efficacy) were evident at both glutamate and GABA synapses for each anesthetic. For example, our results demonstrate that the two barbiturates studied appear to have differing degrees of effect at GABA synapses since thiopental's depressant effects were reversed ~ 65 % by a GABA antagonist, but pentobarbital's effects were only reversed by ~ 55 %. Similarly, these two barbiturates exhibited differing degrees of depression for glutamate-mediated excitatory inputs to the CA1 neurons, pentobarbital produced a 45 % depression in contrast to thiopental with only a 25 % depression. It was interesting that opposite actions were seen at presynaptic sites (GABA release was increased by anesthetics, while glutamate release was depressed) and at postsynaptic sites (GABA-mediated synaptic currents were prolonged, glutamate-mediated currents were not). The MAS theory can readily account for the unique agent-specific profiles of effects observed in various experimental models, and also seen clinically – a long standing weakness of Unitary theories [56]. Finally, the MAS theory predicts that agents which selectively target GABA and glutamate synapses could lead to the design of safer and more effective therapeutic agents for anesthesia, that exhibit fewer undesirable side effects.

Glutamate and GABA synapses in the hippocampus are among the best characterized synapses in the brain and appear to utilize receptor subtypes which are similar to those in neocortex, thalamus and other higher brain regions. Thus, the effects described in the present study would be expected to occur in these other brain regions as well, but it should be noted that different GABA and glutamate receptor subtype distributions are known to occur in cerebellum, spinal and some brain stem nuclei, and it remains to be determined whether anesthetics alter these synapses in a similar manner to their hippocampal counterparts. Ted Eger's group at UCSF has recently found that enhanced GABA-mediated synaptic transmission at the spinal level plays an important role for propofol-induced immobility in response to a noxious stimulus [54], but this was not the case for isoflurane-induced immobility [57]. This agrees well with our findings that the volatile anesthetic-induced depression of synaptic signaling involves mechanisms other than enhanced GABA inhibition (see also [58]), while the depression produced by the barbiturates and propofol are more dependent on enhanced GABA-mediated inhibition. Additional *in vivo *support comes from studies utilizing a GABA beta 3 receptor mutant mouse model – proprofol-induced anesthesia was blocked in these mice, while volatile anesthetic effects were not [59]. Taken together with these *in vivo *findings, our results indicate that effects on GABA synapses play a role in anesthetic actions, especially for propofol, thiopental and pentobarbital; but the results also indicate that effects on glutamate synapses and postsynaptic membrane excitability contribute to the CNS depression produced by all anesthetics. Given the multiple effects observed for anesthetic actions on the two types of synapses studied here, it is likely that effects on other neurotransmitter systems also contribute to anesthetic-induced depression of the CNS.

## Methods

Male Sprague-Dawley rats were anesthetized with ether (22 vol % in air) and the brain was rapidly removed and placed in ice cold (5°C) and pregassed (95/5 % O_2_/CO_2_, carbogen) artificial cerebral spinal fluid (ACSF). The ACSF had the following composition (in mM): Na 151.25; K 2.5; Ca 2.0; Mg 2.0; Cl 131.5; HCO_3 _26.0; SO_4 _2.0; H_2_PO_4 _1.25; and glucose 10. Whole brain coronal slices (450 μm) were cut using a vibratome (Campden Instruments), following careful removal of the dura and pia membranes. Hemisected brain slices were equilibrated for at least one hour at room temperature in an incubation chamber filled with ACSF and continually bubbled with carbogen. Individual slices were transferred to a recording chamber and equilibrated for an additional 10 minutes prior to electrophysiological recording. Oxygenated ACSF solution was continuously perfused through the chamber at a flow rate of 3.0 ml/min and maintained at 22 ± 1°C. The present studies were carried out at room temperature because synaptic responses recorded from cooler brain slices exhibit considerably better baseline stability and the tissue remains viable for many more hours *in vitro *compared to slices maintained at physiological temperatures. Room temperature also facilitates the use of submerged preparations (oxygen solubility and delivery to slices is increased), which allows the use of 60× optics to visualize single neurons for the patch clamp recordings used in some experiments. Previous studies comparing both volatile and intravenous anesthetic effects at physiological and cooler temperatures in brain slices found that there were no apparent differences in effects [43,61,66]. The most important effect of lower temperature is to increase the aqueous solubility of the volatile anesthetics and previous work from our laboratory has described in detail the solubility changes observed at 22 *vs*. 35°C and our methods for measuring and compensating for changed aqueous solubility, as well as the remarkably similar physiological responses recorded from brain slices at these two temperatures [43,66].

To measure population spikes, bipolar tungsten microelectrodes were placed on Schaffer-collateral fibers to electrically stimulate inputs to hippocampal CA1 pyramidal neurons. Glass recording electrodes filled with ACSF (2 to 5 KOhm) were placed in stratum pyramidale to record stimulus-evoked population spike field potentials, or in stratum radiatum to record field EPSPs. Single stimulus pulses (0.01 to 0.05 ms duration; 10 to 80 μA @ 1.0 to 5.0 V) were delivered via constant current isolation units (Grass Instruments, SIU 6D) from a Grass S8800 two channel stimulator; at stimulus rates of 0.05 Hz. Field potential signals were amplified (× 1000), filtered (1 Hz to 10 KHz, bandpass), conditioned (DC offset), and digitally stored for later analysis (A/D with 20 μs resolution on a 486, and 50 MHz microcomputer using Data Wave Systems Corp. or Strathclyde Electrophysiological software).

Whole cell patch-clamp recordings were made using thin-walled borosilicate capillaries (1.5 mm O.D.) pulled in two stages on a Narishige PP83 pipette puller. Patch electrodes were filled with the following intracellular solution (in mM): potassium gluconate or CsCl2 – 100, EGTA – 10, MgCl2 – 5, HEPES free acid – 40, ATP disodium salt – 0.3, and GTP sodium salt -0.3. The electrode solution also contained the local anesthetic QX 314 (1.0 mM) in some experiments, to prevent action potential discharge that would contaminate recordings of IPSCs. Electrode solutions were filtered and pH adjusted to 7.2 using KOH or CsOH and had a final osmolarity of 260 to 270 mOSM. Patch electrodes with a DC resistance of 4 to 5 MOhm were used. Recordings were made using an Axoclamp 2A preamplifier (Axon Instruments) in single electrode voltage clamp mode with > 80 % series resistance compensation and > 5 GOhm seals. Patch-clamp current signals were filtered (0.1 Hz to 10 KHz, bandpass), amplified (× 100) and digitized (10 KHz) for storage and analysis. Frequency and amplitudes of IPSCs were analyzed using Data Wave Technologies and Strathclyde Electrophysiological software in a continuous data recording configuration.

The intravenous anesthetics (propofol and pentobarbital) were made fresh for each experiment, solubilized using 0.5% dimethyl sulfoxide (DMSO) and sonicated immediately prior to test administration in stock solutions and serially diluted into ACSF to achieve the final concentrations for testing. Volatile anesthetics (halothane and isoflurane) were applied in the perfusate at equilibrated concentrations, delivered from calibrated vaporizers and bubbled into the perfusate for at least 10 min prior to switching from control ACSF, to ensure steady-state concentrations were achieved. The concentration of volatile anesthetics in the gas phase were continually measured using a Puritan-Bennett anesthetic monitor. Only a single concentration of a given anesthetic was tested on each brain slice.

Data are expressed as the mean ± standard deviation and statistical analysis (ANOVA with post Tukey test) was performed using Instat from GraphPad Software. For drug effects on paired pulse inhibition, the percent change was first calculated as: Drug 1st/Control 1st = (0.5 × 100) - 100 % = 50 % depression; and Drug 2nd/Control 2nd = (X × 100) - 100 % = X % depression. Then the percent increase in paired pulse inhibition was = (X / 50) × 100 %. This approach has the advantage of normalizing paired responses with respect to varying degrees of population spike facilitation observed during control recordings, i.e. differing degrees of EPSP facilitation on a background of differing degrees of inhibition from preparation to preparation.

## Authors' contributions

SP and AMH contributed equally to this study by conducting most of the experimental work and they also contributed to data analysis and helped with figure preparation. MBM conceived of the study and contributed to experimental design, data analysis and interpretation of results; as well as wrote and prepared the manuscript.

## Figures and Tables

**Table 1:** GABA antagonist effects on anesthetic-induced depression of population spike responses

Anesthetic	Percent reversal of anesthetic-induced depression		
	Bicuculline	Pictrotoxin	CGP-GABA_B_

Propofol	69.5 ± 14.3 %	72.3 ± 8.2 %	3.1 ± 4.1%
Thiopental	64.9 ± 12.9 %	68.3 ± 9.7 %	1.3 ± 3.0%
Pentobarbital	56.2 ± 12.4 %	54.3 ± 11. 5%	0.8 ± 6.3 %
Halothane	22.3 ± 18.4 %	20.8 ± 15.3 %	0.5 ± 3.3 %
Isoflurane	16.2 ± 7.4 %	19.5 ± 10.2 %	3.4 ± 4.8 %

Notes: Bicuculline effects on propofol, thiopental and pentobarbital p < 0.01

Bicuculline effects on halothane and isoflurane p < 0.1

Picrotoxin effects on propofol, thiopental and pentobarbital p < 0.01

Picrotoxin effects on halothane and isoflurane p < 0.1

CGP effects were not significant for any anesthetic studied.
